# 3-(1-Hydr­oxy-2-phenyl­prop-2-en-1-yl)phenol

**DOI:** 10.1107/S1600536810012018

**Published:** 2010-04-10

**Authors:** Ignez Caracelli, Julio Zukerman-Schpector, Fateh V. Singh, Hélio A. Stefani, Edward R. T. Tiekink

**Affiliations:** a BioMat-Physics Department, Univ Estadual Paulista, UNESP, 17033-360 Bauru, SP, Brazil; b Department of Chemistry, Universidade Federal de São Carlos, 13565-905 São Carlos, SP, Brazil; c Departamento de Farmácia, Faculdade de Ciências Farmacêuticas, Universidade de São Paulo, São Paulo-SP, Brazil; d Department of Chemistry, University of Malaya, Kuala Lumpur 50603, Malaysia

## Abstract

Two independent pseudo-enanti­omeric mol­ecules comprise the asymmetric unit in the title compound, C_15_H_14_O_2_. While the central O—C—C—C residue approaches planarity [torsion angles = −15.8 (3) (mol­ecule *a*) and 15.4 (3)° (mol­ecule *b*)], the benzene rings are approximately orthogonal [the dihedral angles formed between the benzene rings are 62.89 (12) (mol­ecule *a*) and 80.15 (12)° (mol­ecule *b*)]. Two-dimensional arrays in the *ab* plane sustained by O—H⋯O hydrogen bonding are found in the crystal structure.

## Related literature

For the synthesis of the title compound and the motivation for its study, see: Singh *et al.* (2010 ▶).
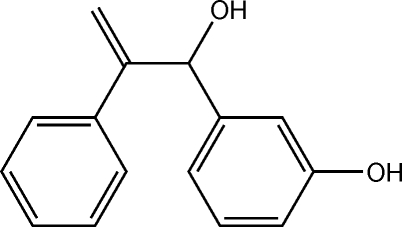


## Experimental

### 

#### Crystal data

C_15_H_14_O_2_
*M* *_r_* = 226.28
Orthorhombic, 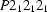

*a* = 9.1301 (2) Å
*b* = 10.2026 (2) Å
*c* = 24.8379 (6) Å
*V* = 2313.67 (9) Å^3^
*Z* = 8
Mo *K*α radiation
μ = 0.09 mm^−1^
*T* = 100 K
0.27 × 0.13 × 0.13 mm

#### Data collection

Bruker SMART APEXII diffractometer
Absorption correction: multi-scan (*SADABS*; Sheldrick, 1996 ▶) *T*_min_ = 0.883, *T*_max_ = 1
31791 measured reflections
2368 independent reflections
2150 reflections with *I* > 2σ(*I*)
*R*_int_ = 0.042

#### Refinement

*R*[*F* ^2^ > 2σ(*F* ^2^)] = 0.035
*wR*(*F* ^2^) = 0.084
*S* = 1.15
2368 reflections
311 parameters
H-atom parameters constrained
Δρ_max_ = 0.17 e Å^−3^
Δρ_min_ = −0.15 e Å^−3^

### 

Data collection: *APEX2* (Bruker, 2007 ▶); cell refinement: *SAINT* (Bruker, 2007 ▶); data reduction: *SAINT*; program(s) used to solve structure: *SIR97* (Altomare *et al.*, 1999 ▶); program(s) used to refine structure: *SHELXL97* (Sheldrick, 2008 ▶); molecular graphics: *ORTEP-3* (Farrugia, 1997 ▶), *DIAMOND* (Brandenburg, 2006 ▶) and *MarvinSketch* (Chemaxon, 2009 ▶); software used to prepare material for publication: *publCIF* (Westrip, 2010 ▶).

## Supplementary Material

Crystal structure: contains datablocks global, I. DOI: 10.1107/S1600536810012018/hg2665sup1.cif

Structure factors: contains datablocks I. DOI: 10.1107/S1600536810012018/hg2665Isup2.hkl

Additional supplementary materials:  crystallographic information; 3D view; checkCIF report

## Figures and Tables

**Table 1:** Hydrogen-bond geometry (Å, °)

*D*—H⋯*A*	*D*—H	H⋯*A*	*D*⋯*A*	*D*—H⋯*A*
O1—H1*O*⋯O4	0.84	1.89	2.727 (2)	175
O2—H2*O*⋯O1^i^	0.84	2.00	2.823 (2)	168
O3—H3*O*⋯O2^ii^	0.84	1.89	2.728 (2)	174
O4—H4*O*⋯O3^iii^	0.84	2.02	2.825 (2)	161

Symmetry codes: (i) 
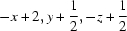
; (ii) 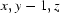; (iii) 
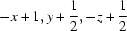
.

**Fig. 1.**
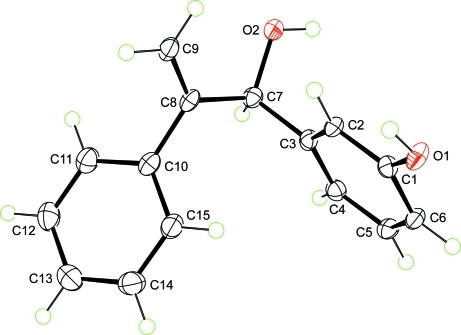
The molecular structure of the first independent molecule in (I) showing atom labelling scheme and displacement ellipsoids at the 50% probability level (arbitrary spheres for the H atoms).

**Fig. 2.**
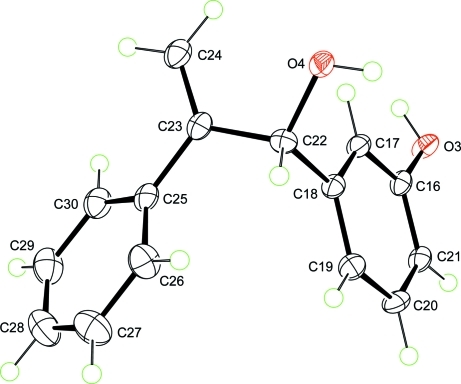
The molecular structure of the second independent molecule in (I) showing atom labelling scheme and displacement ellipsoids at the 50% probability level (arbitrary spheres for the H atoms).

**Fig. 3.**
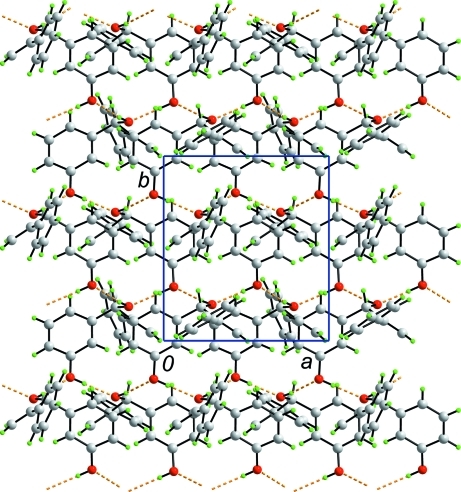
The supramolecular arrangement showing the formation of two-dimensional arrays in the ab plane.

**Fig. 4.**
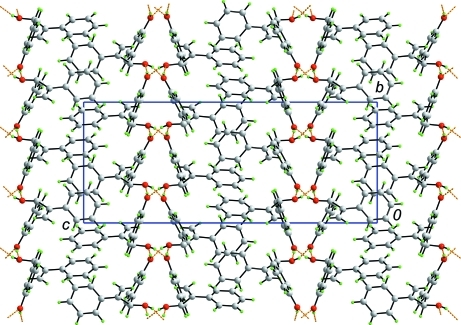
The stacking of the two-dimensional arrays along the c axis.

## supplementary crystallographic information

### Comment

The title compound, (I), was prepared in connection with a study of the synthesis of α,β-epoxy ketones using a palladium-catalyzed epoxidation-oxidation sequence (Singh *et al.*, 2010). Two independent molecules, molecule *a* (Fig. 1) and molecule *b* (Fig. 2), comprise the crystallographic asymmetric unit. Molecules *a* and *b* are related by a non-crystallographic centre of inversion. Close intramolecular O2···H9b and O4···H24b contacts which close S(6) motifs are noted, Table 1. These interactions are probably responsible for the near planarity of the O2–C7–C8–C9 and O4–C22–C23–C24 residues as seen in the respective torsion angles of -15.8 (3) and 15.4 (3)°. The benzene rings are approximately orthogonal [the dihedral angles formed between the benzene rings is 62.89 (12) ° (molecule *a*) and 80.15 (12) ° (molecule *b*)].

In the crystal packing, O–H···O interactions predominate, Table 1, and lead to the formation of two-dimensional arrays in the *ab* plane, Fig. 3, that stack along the *c* axis, Fig. 4.

### Experimental

The synthesis was described in Singh *et al.* (2010) and crystals were grown by slow evaporation from a solution of 15% of acetyl acetate in hexane.

### Refinement

The H atoms were geometrically placed (O–H = 0.84 Å and C–H = 0.95–1.00 Å) and refined as riding with *U_iso_*(H) = 1.2*U_eq_*(C) and *U_iso_*(H) = 1.5*U_eq_*(O). In the absence of significant anomalous scattering effects, 1752 Friedel pairs were averaged in the final refinement.

### Crystal data

**Table:**

C_15_H_14_O_2_	*F*(000) = 960
*M**_r_* = 226.28	*D*_x_ = 1.299 Mg m^−^^3^
Orthorhombic, *P*2_1_2_1_2_1_	Mo *K*α radiation, λ = 0.71073 Å
Hall symbol: P 2ac 2ab	Cell parameters from 9974 reflections
*a* = 9.1301 (2) Å	θ = 2.5–25.0°
*b* = 10.2026 (2) Å	µ = 0.09 mm^−^^1^
*c* = 24.8379 (6) Å	*T* = 100 K
*V* = 2313.67 (9) Å^3^	Block, colourless
*Z* = 8	0.27 × 0.13 × 0.13 mm

### Data collection

**Table:**

Bruker SMART APEXII diffractometer	2368 independent reflections
Radiation source: sealed tube	2150 reflections with *I* > 2σ(*I*)
graphite	*R*_int_ = 0.042
φ and ω scans	θ_max_ = 25.1°, θ_min_ = 1.6°
Absorption correction: multi-scan (*SADABS*; Sheldrick, 1996)	*h* = −10→10
*T*_min_ = 0.883, *T*_max_ = 1	*k* = −12→11
31791 measured reflections	*l* = −29→29

### Refinement

**Table:**

Refinement on *F*^2^	Primary atom site location: structure-invariant direct methods
Least-squares matrix: full	Secondary atom site location: difference Fourier map
*R*[*F*^2^ > 2σ(*F*^2^)] = 0.035	Hydrogen site location: inferred from neighbouring sites
*wR*(*F*^2^) = 0.084	H-atom parameters constrained
*S* = 1.15	*w* = 1/[σ^2^(*F*_o_^2^) + (0.0381*P*)^2^ + 0.6989*P*] where *P* = (*F*_o_^2^ + 2*F*_c_^2^)/3
2368 reflections	(Δ/σ)_max_ < 0.001
311 parameters	Δρ_max_ = 0.17 e Å^−^^3^
0 restraints	Δρ_min_ = −0.15 e Å^−^^3^

### Special details

**Table:**

Geometry. All s.u.'s (except the s.u. in the dihedral angle between two l.s. planes) are estimated using the full covariance matrix. The cell s.u.'s are taken into account individually in the estimation of s.u.'s in distances, angles and torsion angles; correlations between s.u.'s in cell parameters are only used when they are defined by crystal symmetry. An approximate (isotropic) treatment of cell s.u.'s is used for estimating s.u.'s involving l.s. planes.
Refinement. Refinement of *F*^2^ against ALL reflections. The weighted *R*-factor wR and goodness of fit *S* are based on *F*^2^, conventional *R*-factors *R* are based on *F*, with *F* set to zero for negative *F*^2^. The threshold expression of *F*^2^ > σ(*F*^2^) is used only for calculating *R*-factors(gt) etc. and is not relevant to the choice of reflections for refinement. *R*-factors based on *F*^2^ are statistically about twice as large as those based on *F*, and *R*- factors based on ALL data will be even larger.

### Fractional atomic coordinates and isotropic or equivalent isotropic displacement parameters (Å^2^)

**Table:**

	*x*	*y*	*z*	*U*_iso_*/*U*_eq_	
O1	1.06075 (18)	0.29142 (16)	0.21652 (7)	0.0217 (4)	
H1O	0.9766	0.2581	0.2163	0.033*	
O2	0.70773 (18)	0.69539 (17)	0.21929 (6)	0.0203 (4)	
H2O	0.7719	0.7160	0.2420	0.031*	
C1	1.0535 (3)	0.4174 (2)	0.19749 (8)	0.0177 (5)	
C2	0.9221 (3)	0.4834 (3)	0.19232 (9)	0.0177 (5)	
H2	0.8332	0.4418	0.2023	0.021*	
C3	0.9201 (3)	0.6112 (2)	0.17244 (9)	0.0169 (5)	
C4	1.0502 (3)	0.6719 (3)	0.15824 (9)	0.0189 (5)	
H4	1.0492	0.7590	0.1447	0.023*	
C5	1.1816 (3)	0.6054 (2)	0.16377 (9)	0.0194 (5)	
H5	1.2708	0.6474	0.1541	0.023*	
C6	1.1841 (3)	0.4781 (3)	0.18328 (9)	0.0194 (5)	
H6	1.2744	0.4327	0.1869	0.023*	
C7	0.7742 (2)	0.6817 (2)	0.16736 (9)	0.0176 (5)	
H7	0.7917	0.7710	0.1520	0.021*	
C8	0.6701 (3)	0.6085 (2)	0.13080 (9)	0.0177 (5)	
C9	0.5564 (3)	0.5408 (2)	0.14915 (10)	0.0204 (5)	
H9A	0.4929	0.4973	0.1247	0.025*	
H9B	0.5384	0.5359	0.1868	0.025*	
C10	0.7000 (3)	0.6172 (2)	0.07155 (9)	0.0182 (5)	
C11	0.6005 (3)	0.6817 (3)	0.03877 (10)	0.0260 (6)	
H11	0.5163	0.7213	0.0543	0.031*	
C12	0.6225 (3)	0.6892 (3)	−0.01631 (10)	0.0288 (6)	
H12	0.5540	0.7345	−0.0383	0.035*	
C13	0.7432 (3)	0.6313 (3)	−0.03926 (10)	0.0270 (6)	
H13	0.7582	0.6362	−0.0771	0.032*	
C14	0.8429 (3)	0.5659 (3)	−0.00699 (10)	0.0286 (6)	
H14	0.9257	0.5247	−0.0228	0.034*	
C15	0.8223 (3)	0.5602 (3)	0.04816 (10)	0.0242 (6)	
H15	0.8925	0.5171	0.0701	0.029*	
O3	0.43410 (18)	−0.19891 (17)	0.22734 (7)	0.0221 (4)	
H3O	0.5183	−0.2321	0.2274	0.033*	
O4	0.78763 (17)	0.18479 (17)	0.20933 (6)	0.0200 (4)	
H4O	0.7310	0.2091	0.2341	0.030*	
C16	0.4372 (3)	−0.0816 (2)	0.20043 (9)	0.0171 (5)	
C17	0.5664 (3)	−0.0131 (2)	0.19299 (9)	0.0186 (5)	
H17	0.6560	−0.0481	0.2061	0.022*	
C18	0.5656 (3)	0.1067 (2)	0.16646 (9)	0.0168 (5)	
C19	0.4346 (3)	0.1569 (2)	0.14687 (9)	0.0205 (5)	
H19	0.4335	0.2381	0.1282	0.025*	
C20	0.3058 (3)	0.0881 (2)	0.15472 (9)	0.0208 (5)	
H20	0.2162	0.1228	0.1415	0.025*	
C21	0.3063 (3)	−0.0308 (3)	0.18165 (9)	0.0188 (5)	
H21	0.2173	−0.0772	0.1872	0.023*	
C22	0.7091 (3)	0.1805 (2)	0.15946 (9)	0.0180 (5)	
H22	0.6873	0.2722	0.1476	0.022*	
C23	0.8034 (3)	0.1145 (2)	0.11719 (9)	0.0181 (5)	
C24	0.9210 (3)	0.0451 (3)	0.13067 (10)	0.0237 (6)	
H24A	0.9762	0.0015	0.1036	0.028*	
H24B	0.9499	0.0391	0.1673	0.028*	
C25	0.7532 (3)	0.1273 (2)	0.06044 (9)	0.0198 (5)	
C26	0.6886 (3)	0.2420 (3)	0.04143 (10)	0.0291 (6)	
H26	0.6748	0.3136	0.0653	0.035*	
C27	0.6439 (3)	0.2536 (3)	−0.01169 (11)	0.0367 (7)	
H27	0.5991	0.3323	−0.0239	0.044*	
C28	0.6648 (3)	0.1505 (3)	−0.04687 (11)	0.0349 (7)	
H28	0.6359	0.1585	−0.0835	0.042*	
C29	0.7273 (3)	0.0368 (3)	−0.02884 (10)	0.0325 (7)	
H29	0.7414	−0.0342	−0.0530	0.039*	
C30	0.7702 (3)	0.0245 (3)	0.02443 (10)	0.0258 (6)	
H30	0.8119	−0.0556	0.0365	0.031*	

### Atomic displacement parameters (Å^2^)

**Table:**

	*U*^11^	*U*^22^	*U*^33^	*U*^12^	*U*^13^	*U*^23^
O1	0.0130 (8)	0.0196 (9)	0.0326 (9)	−0.0014 (8)	−0.0031 (8)	0.0047 (8)
O2	0.0157 (9)	0.0261 (9)	0.0192 (8)	0.0022 (8)	−0.0011 (7)	−0.0020 (8)
C1	0.0173 (12)	0.0196 (13)	0.0163 (11)	−0.0006 (11)	−0.0021 (10)	−0.0016 (10)
C2	0.0105 (11)	0.0241 (14)	0.0186 (11)	−0.0027 (11)	−0.0003 (9)	−0.0010 (10)
C3	0.0148 (12)	0.0191 (13)	0.0167 (11)	0.0003 (11)	−0.0027 (9)	−0.0034 (10)
C4	0.0194 (12)	0.0205 (14)	0.0169 (11)	−0.0013 (11)	−0.0009 (10)	−0.0009 (10)
C5	0.0140 (12)	0.0239 (14)	0.0205 (11)	−0.0079 (11)	0.0009 (10)	−0.0031 (11)
C6	0.0120 (12)	0.0251 (14)	0.0212 (11)	0.0015 (11)	−0.0011 (10)	−0.0029 (10)
C7	0.0157 (12)	0.0172 (12)	0.0197 (11)	−0.0014 (11)	−0.0002 (9)	0.0015 (10)
C8	0.0124 (12)	0.0159 (13)	0.0248 (12)	0.0032 (11)	−0.0013 (10)	−0.0007 (10)
C9	0.0142 (12)	0.0235 (14)	0.0236 (12)	−0.0002 (11)	−0.0026 (10)	−0.0011 (11)
C10	0.0151 (12)	0.0131 (12)	0.0263 (12)	−0.0042 (11)	−0.0008 (10)	−0.0006 (10)
C11	0.0214 (14)	0.0273 (14)	0.0293 (13)	0.0040 (12)	−0.0024 (11)	0.0007 (12)
C12	0.0275 (15)	0.0305 (15)	0.0283 (13)	0.0014 (13)	−0.0069 (12)	0.0050 (12)
C13	0.0319 (15)	0.0264 (14)	0.0226 (12)	−0.0094 (13)	0.0003 (11)	0.0001 (11)
C14	0.0243 (14)	0.0334 (16)	0.0282 (13)	−0.0034 (13)	0.0043 (11)	−0.0058 (12)
C15	0.0186 (13)	0.0274 (14)	0.0266 (12)	0.0011 (12)	−0.0021 (11)	−0.0010 (11)
O3	0.0142 (9)	0.0208 (9)	0.0313 (9)	0.0014 (8)	0.0024 (7)	0.0046 (8)
O4	0.0149 (8)	0.0252 (9)	0.0198 (8)	−0.0019 (8)	0.0012 (7)	−0.0015 (7)
C16	0.0157 (12)	0.0163 (12)	0.0194 (11)	−0.0012 (11)	0.0025 (10)	−0.0034 (10)
C17	0.0137 (12)	0.0216 (14)	0.0204 (11)	0.0005 (11)	−0.0003 (10)	−0.0025 (10)
C18	0.0143 (12)	0.0183 (13)	0.0178 (11)	−0.0012 (11)	−0.0001 (9)	−0.0036 (10)
C19	0.0198 (13)	0.0191 (14)	0.0227 (12)	0.0015 (11)	0.0001 (10)	−0.0006 (10)
C20	0.0123 (12)	0.0244 (14)	0.0255 (12)	−0.0001 (11)	−0.0031 (10)	−0.0018 (11)
C21	0.0136 (12)	0.0216 (13)	0.0211 (11)	−0.0045 (11)	0.0017 (10)	−0.0037 (10)
C22	0.0181 (12)	0.0167 (12)	0.0192 (11)	−0.0018 (11)	−0.0024 (10)	0.0012 (10)
C23	0.0146 (12)	0.0163 (12)	0.0235 (11)	−0.0056 (11)	0.0025 (10)	−0.0004 (10)
C24	0.0175 (13)	0.0263 (14)	0.0273 (13)	−0.0031 (12)	0.0021 (11)	−0.0024 (11)
C25	0.0137 (12)	0.0219 (13)	0.0239 (12)	−0.0058 (11)	0.0033 (10)	0.0012 (10)
C26	0.0357 (16)	0.0253 (14)	0.0262 (13)	−0.0008 (13)	0.0011 (12)	−0.0013 (11)
C27	0.0452 (19)	0.0330 (16)	0.0318 (15)	−0.0030 (14)	−0.0044 (13)	0.0088 (14)
C28	0.0398 (17)	0.0419 (18)	0.0229 (13)	−0.0148 (15)	−0.0025 (13)	0.0022 (12)
C29	0.0343 (16)	0.0360 (16)	0.0272 (13)	−0.0089 (14)	0.0029 (12)	−0.0085 (12)
C30	0.0230 (14)	0.0271 (14)	0.0273 (13)	−0.0030 (12)	0.0024 (11)	−0.0026 (11)

### Geometric parameters (Å, °)

**Table:**

O1—C1	1.371 (3)	O3—C16	1.371 (3)
O1—H1O	0.8400	O3—H3O	0.8400
O2—C7	1.432 (3)	O4—C22	1.432 (3)
O2—H2O	0.8400	O4—H4O	0.8400
C1—C2	1.382 (3)	C16—C21	1.383 (3)
C1—C6	1.389 (4)	C16—C17	1.384 (3)
C2—C3	1.394 (4)	C17—C18	1.389 (3)
C2—H2	0.9500	C17—H17	0.9500
C3—C4	1.385 (3)	C18—C19	1.388 (3)
C3—C7	1.520 (3)	C18—C22	1.521 (3)
C4—C5	1.386 (3)	C19—C20	1.383 (3)
C4—H4	0.9500	C19—H19	0.9500
C5—C6	1.387 (4)	C20—C21	1.386 (4)
C5—H5	0.9500	C20—H20	0.9500
C6—H6	0.9500	C21—H21	0.9500
C7—C8	1.512 (3)	C22—C23	1.516 (3)
C7—H7	1.0000	C22—H22	1.0000
C8—C9	1.327 (3)	C23—C24	1.329 (3)
C8—C10	1.499 (3)	C23—C25	1.488 (3)
C9—H9A	0.9500	C24—H24A	0.9500
C9—H9B	0.9500	C24—H24B	0.9500
C10—C11	1.386 (3)	C25—C30	1.388 (3)
C10—C15	1.387 (3)	C25—C26	1.393 (4)
C11—C12	1.385 (4)	C26—C27	1.386 (4)
C11—H11	0.9500	C26—H26	0.9500
C12—C13	1.374 (4)	C27—C28	1.381 (4)
C12—H12	0.9500	C27—H27	0.9500
C13—C14	1.384 (4)	C28—C29	1.368 (4)
C13—H13	0.9500	C28—H28	0.9500
C14—C15	1.384 (3)	C29—C30	1.385 (4)
C14—H14	0.9500	C29—H29	0.9500
C15—H15	0.9500	C30—H30	0.9500
			
C1—O1—H1O	109.5	C16—O3—H3O	109.5
C7—O2—H2O	109.5	C22—O4—H4O	109.5
O1—C1—C2	122.1 (2)	O3—C16—C21	118.3 (2)
O1—C1—C6	117.6 (2)	O3—C16—C17	121.6 (2)
C2—C1—C6	120.3 (2)	C21—C16—C17	120.1 (2)
C1—C2—C3	120.0 (2)	C16—C17—C18	120.2 (2)
C1—C2—H2	120.0	C16—C17—H17	119.9
C3—C2—H2	120.0	C18—C17—H17	119.9
C4—C3—C2	119.8 (2)	C19—C18—C17	119.7 (2)
C4—C3—C7	121.3 (2)	C19—C18—C22	121.3 (2)
C2—C3—C7	118.9 (2)	C17—C18—C22	119.0 (2)
C3—C4—C5	119.9 (2)	C20—C19—C18	119.7 (2)
C3—C4—H4	120.0	C20—C19—H19	120.1
C5—C4—H4	120.0	C18—C19—H19	120.1
C4—C5—C6	120.5 (2)	C19—C20—C21	120.6 (2)
C4—C5—H5	119.8	C19—C20—H20	119.7
C6—C5—H5	119.8	C21—C20—H20	119.7
C5—C6—C1	119.5 (2)	C16—C21—C20	119.6 (2)
C5—C6—H6	120.3	C16—C21—H21	120.2
C1—C6—H6	120.3	C20—C21—H21	120.2
O2—C7—C8	108.83 (18)	O4—C22—C23	109.16 (19)
O2—C7—C3	110.03 (18)	O4—C22—C18	110.34 (18)
C8—C7—C3	111.52 (19)	C23—C22—C18	110.42 (19)
O2—C7—H7	108.8	O4—C22—H22	109.0
C8—C7—H7	108.8	C23—C22—H22	109.0
C3—C7—H7	108.8	C18—C22—H22	109.0
C9—C8—C10	120.7 (2)	C24—C23—C25	122.3 (2)
C9—C8—C7	122.8 (2)	C24—C23—C22	121.4 (2)
C10—C8—C7	116.5 (2)	C25—C23—C22	116.2 (2)
C8—C9—H9A	120.0	C23—C24—H24A	120.0
C8—C9—H9B	120.0	C23—C24—H24B	120.0
H9A—C9—H9B	120.0	H24A—C24—H24B	120.0
C11—C10—C15	118.7 (2)	C30—C25—C26	117.7 (2)
C11—C10—C8	119.1 (2)	C30—C25—C23	120.6 (2)
C15—C10—C8	122.2 (2)	C26—C25—C23	121.7 (2)
C12—C11—C10	120.8 (2)	C27—C26—C25	121.3 (3)
C12—C11—H11	119.6	C27—C26—H26	119.4
C10—C11—H11	119.6	C25—C26—H26	119.4
C13—C12—C11	120.1 (2)	C28—C27—C26	119.8 (3)
C13—C12—H12	119.9	C28—C27—H27	120.1
C11—C12—H12	119.9	C26—C27—H27	120.1
C12—C13—C14	119.6 (2)	C29—C28—C27	119.8 (2)
C12—C13—H13	120.2	C29—C28—H28	120.1
C14—C13—H13	120.2	C27—C28—H28	120.1
C15—C14—C13	120.3 (3)	C28—C29—C30	120.5 (3)
C15—C14—H14	119.9	C28—C29—H29	119.7
C13—C14—H14	119.9	C30—C29—H29	119.7
C14—C15—C10	120.4 (2)	C29—C30—C25	121.0 (3)
C14—C15—H15	119.8	C29—C30—H30	119.5
C10—C15—H15	119.8	C25—C30—H30	119.5
			
O1—C1—C2—C3	−179.59 (19)	O3—C16—C17—C18	−178.75 (19)
C6—C1—C2—C3	0.4 (3)	C21—C16—C17—C18	−0.2 (3)
C1—C2—C3—C4	−0.4 (3)	C16—C17—C18—C19	−0.7 (3)
C1—C2—C3—C7	−179.8 (2)	C16—C17—C18—C22	179.3 (2)
C2—C3—C4—C5	0.1 (3)	C17—C18—C19—C20	1.1 (3)
C7—C3—C4—C5	179.4 (2)	C22—C18—C19—C20	−179.0 (2)
C3—C4—C5—C6	0.3 (3)	C18—C19—C20—C21	−0.4 (4)
C4—C5—C6—C1	−0.2 (3)	O3—C16—C21—C20	179.43 (19)
O1—C1—C6—C5	179.91 (19)	C17—C16—C21—C20	0.9 (3)
C2—C1—C6—C5	−0.1 (3)	C19—C20—C21—C16	−0.5 (3)
C4—C3—C7—O2	−117.5 (2)	C19—C18—C22—O4	132.6 (2)
C2—C3—C7—O2	61.8 (3)	C17—C18—C22—O4	−47.4 (3)
C4—C3—C7—C8	121.6 (2)	C19—C18—C22—C23	−106.6 (2)
C2—C3—C7—C8	−59.0 (3)	C17—C18—C22—C23	73.3 (3)
O2—C7—C8—C9	−15.8 (3)	O4—C22—C23—C24	15.4 (3)
C3—C7—C8—C9	105.8 (3)	C18—C22—C23—C24	−106.1 (3)
O2—C7—C8—C10	163.3 (2)	O4—C22—C23—C25	−167.12 (19)
C3—C7—C8—C10	−75.2 (3)	C18—C22—C23—C25	71.4 (3)
C9—C8—C10—C11	65.5 (3)	C24—C23—C25—C30	33.9 (4)
C7—C8—C10—C11	−113.6 (3)	C22—C23—C25—C30	−143.6 (2)
C9—C8—C10—C15	−113.0 (3)	C24—C23—C25—C26	−145.8 (3)
C7—C8—C10—C15	67.9 (3)	C22—C23—C25—C26	36.8 (3)
C15—C10—C11—C12	0.1 (4)	C30—C25—C26—C27	−0.6 (4)
C8—C10—C11—C12	−178.5 (2)	C23—C25—C26—C27	179.1 (3)
C10—C11—C12—C13	0.6 (4)	C25—C26—C27—C28	−0.7 (4)
C11—C12—C13—C14	−0.2 (4)	C26—C27—C28—C29	1.1 (4)
C12—C13—C14—C15	−0.9 (4)	C27—C28—C29—C30	−0.2 (4)
C13—C14—C15—C10	1.6 (4)	C28—C29—C30—C25	−1.1 (4)
C11—C10—C15—C14	−1.2 (4)	C26—C25—C30—C29	1.4 (4)
C8—C10—C15—C14	177.3 (2)	C23—C25—C30—C29	−178.3 (2)

### Hydrogen-bond geometry (Å, °)

**Table:**

*D*—H···*A*	*D*—H	H···*A*	*D*···*A*	*D*—H···*A*
C9—H9b···O2	0.95	2.39	2.726 (3)	101
C24—H24b···O4	0.95	2.34	2.708 (3)	102
O1—H1O···O4	0.84	1.89	2.727 (2)	175
O2—H2O···O1^i^	0.84	2.00	2.823 (2)	168
O3—H3O···O2^ii^	0.84	1.89	2.728 (2)	174
O4—H4O···O3^iii^	0.84	2.02	2.825 (2)	161

Symmetry codes:  (i) −*x*+2, *y*+1/2, −*z*+1/2; (ii) *x*, *y*−1, *z*; (iii) −*x*+1, *y*+1/2, −*z*+1/2.
